# Trends in cesarean section rates in Brazil by Robson classification group, 2014-2020

**DOI:** 10.1590/0034-7167-2023-0099

**Published:** 2024-07-29

**Authors:** Virginia Barbosa Pereira, Síntia Nascimento dos Reis, Fernanda Gontijo Araújo, Torcata Amorim, Eunice Francisca Martins, Mariana Santos Felisbino-Mendes

**Affiliations:** I Universidade Federal de Minas Gerais. Belo Horizonte, Minas Gerais, Brazil

**Keywords:** Time Serie Studies, Cesarean Section, Maternal Health Services, Reproductive Health, Nursing, Obstetric, Estudios de Series Temporales, Cesárea, Servicios de Salud Materna, Salud Reproductiva, Enfermería Obstétrica

## Abstract

**Objectives:**

to evaluate the trends in cesarean sections from 2014 to 2020 across both public and private sectors, utilizing the Robson Classification.

**Methods:**

this time series study analyzed the proportion of women who underwent cesarean sections between 2014 and 2020, considering both the Robson classification and the type of healthcare service. Trend analysis was conducted using the Prais-Winsten regression.

**Results:**

higher proportions of cesarean sections were observed in all Robson groups within the private sector compared to the public sector. This was despite a decreasing trend in the private sector and an increasing trend in the public sector. Notably, elevated proportions of cesarean sections were recorded in groups that are typically favorable to normal childbirth (Robson 1, 4, and 5).

**Conclusions:**

although there was a decreasing trend in cesarean sections within the private sector, an increasing trend was observed in the public sector. Additionally, there was a high proportion of cesarean sections among women with conditions favorable to normal childbirth. It is crucial to continuously monitor these indicators to evaluate and implement interventions aimed at reducing unnecessary cesarean sections.

## RESUMEN

**Objetivos:**

evaluar la tendencia de las cesáreas, en el período de 2014 a 2020, en los sectores público y privado según la Clasificación de Robson.

**Métodos:**

estudio de serie temporal de la proporción de mujeres que tuvieron cesáreas entre 2014 y 2020, considerando la clasificación de Robson y el tipo de servicio. Para el análisis de tendencia, se utilizó la regresión de Prais-Winsten.

**Resultados:**

se observaron mayores proporciones de cesáreas en todos los grupos de Robson en el sector privado en comparación con el público, incluso con una tendencia a la reducción en el privado y un aumento en el público. También se registraron proporciones elevadas de cesáreas en grupos favorables al parto normal (Robson 1, 4 y 5).

**Conclusiones:**

a pesar de la tendencia a la reducción de las cesáreas en el sector privado, hubo una tendencia creciente en el público y una elevada proporción de cesáreas en mujeres con condiciones favorables al parto normal. Se destaca la necesidad de monitorear estos indicadores para evaluar y proponer intervenciones para la reducción de cesáreas innecesarias.

## INTRODUCTION

Cesarean section is a surgical intervention necessary in specific cases, such as placental abruption, uterine rupture, chronic fetal distress, and other obstetric emergencies, and can even save lives^(1)^. However, the prevalence of obstetric emergencies does not align with the frequency of this surgery, as the majority of women are in healthy condition for vaginal delivery^(2)^. When improperly indicated, a cesarean can cause infections, neonatal respiratory complications, and increase the risk of maternal and fetal death^(2)^. It is also associated with a high rate of iatrogenic prematurity^(3)^.

The World Health Organization (WHO) recommends that the cesarean rate should not exceed 15%, and since 1985, the ideal rate has been considered to be between 10 and 15%^(4)^. The global average cesarean rate is 21.1%, ranging from 5% in Sub-Saharan Africa to 42.8% in Latin America/Caribbean^(5)^. Moreover, there has been a steady increase in cesarean rates worldwide over the last few decades, especially in lowand middle-income countries (LMICs)^(6)^. Brazil has the second-highest cesarean rate in the world (55.7% in 2018), followed by the Dominican Republic (58.1% in 2018)^(7)^. It is noteworthy that nearly 90% of cesareans in Brazil are performed on women who receive healthcare during delivery in private services^(8)^.

Consequently, given the high rate of unnecessary cesareans in the country, various public policies aimed at reducing these rates and oriented toward improving care for pregnant women based on scientific evidence have been implemented over the years. Notable strategies include the Prenatal and Birth Humanization Program of 2002^(9)^; the Stork Network, launched and implemented in the Unified Health System (SUS) in 2011^(10)^; and the Obstetric Nursing Residency Program, established in 2012^(11)^, which was designed to train nurses to work in prenatal care, childbirth, and birth. The WHO has recognized this professional role as capable of contributing to the reduction of unnecessary interventions during childbirth and birth, with their care associated with positive maternal and neonatal outcomes^(3)^. The performance of these nurses is also linked to a lower rate of cesareans^(12-13)^.

In this context of strengthening policies for childbirth and birth care, in 2016, the Guidelines for the Care of Pregnant Women: Cesarean Operation were published, establishing fundamental parameters for guidance and scientific evaluation and addressing the necessity (or lack thereof) of performing a cesarean, as formulated by the General Coordination of Women’s Health of the Ministry of Health^(14)^. Similarly, in 2017, the Ministry of Health’s Ordinance 353 established the National Guidelines for Assistance to Normal Birth, aimed at developing this service with maximum safety and quality, both for women and newborns^(15)^. In the context of mobilization for the reduction of cesareans, in 2016, the National Health Surveillance Agency introduced the Adequate Birth Project with mechanisms to reduce cesareans in private institutions^(16)^.

One method of assessing cesarean rates is the Robson Classification, proposed by the WHO in 2014. This standard instrument enables comparisons between institutions and countries^(4,17)^. The classification includes 10 mutually exclusive and collectively exhaustive groups, ensuring that each pregnant woman can be categorized into only one group, based on six obstetric characteristics: previous parity, prior cesarean, number of fetuses, gestational age, onset of labor, and fetal presentation^(18)^. Thus, this classification facilitates the monitoring and evaluation of cesarean rates across different groups and identifies areas for improvement^(17-18)^.

Studies published on the Robson Classification groups cover various aspects. One study conducted in Austria compared changes in cesarean rates at a university hospital after implementing the Robson Classification, finding that the major contributors to the cesarean rate were multiparous women with term fetuses and previous cesareans^(19)^. Another study assessed the use of the Robson Classification in 21 countries with varying Human Development Index (HDI) levels, demonstrating that the cesarean rate after labor induction in multiparous women significantly increased across all analyzed groups^(17)^ and was higher among women with prior cesareans in countries with moderate and low HDI^(17)^. In Brazil, a study showed that more than 54% of all cesareans were performed before the onset of labor, and the higher the HDI, the higher the cesarean rate among the most vulnerable socioeconomic groups^(20)^.

Additionally, other Brazilian studies using the Robson Classification were localized^(21-22)^, restricted to specific institutions^(23-24)^, or had different objectives, such as evaluating the association between cesareans and prematurity^(25)^ or the association between access to prenatal care and the occurrence of cesareans^(26)^. Other studies employed the Robson Classification to assess the occurrence of cesareans in the country but during periods close to its implementation as a strategy, such as from 2011 to 2017^(20)^, and from 2014 to 2016^(27)^. Furthermore, a study analyzed the temporal trend of cesarean rates from 1994 to 2019, revealing an annual increase of 2.1% and a trend toward stabilization beginning in 2012, alongside regional differences; however, this study did not utilize the Robson Classification^(5)^. Therefore, the importance of analyzing the proportion of cesareans in the country from the moment the WHO recommended the Robson Classification as a tool for monitoring and reducing cesareans^(4)^ is underscored, as well as the separate evaluation of public and private services, given that a higher proportion of cesareans is observed in the country’s private services^(8)^.

In this context of high cesarean rates and disparities among socioeconomic groups, it becomes important to evaluate how the behavior of cesarean rates in the country has evolved, given that there are public policies aimed at reducing them in both public and private hospitals. The Robson Classification is an important tool for monitoring the occurrence of unnecessary cesareans.

## OBJECTIVES

To evaluate the trends in cesarean sections from 2014 to 2020 in both the public and private sectors, according to the Robson Classification.

## METHODS

### Ethical aspects

This study utilized secondary, aggregated public domain data without individual subject identification. Therefore, the requirement for Ethics Committee review was waived, in accordance with National Health Council Ordinance No. 466, dated December 12, 2012^(28)^, and the Informed Consent Form was not required.

### Design, period, and location of the study

This was an ecological time-series study of cesarean rates within public and private health services across the country, following the Robson Classification and adhering to the STROBE guidelines available on the Equator platform^(29)^. Data on pregnancies and births from 2014 to 2019 were extracted from the Department of Informatics of the Unified Health System (DATASUS)^(30)^. The Live Birth Information Systemis one of the systems that feeds into the DATASUS platform and provides data on births in Brazil through the Live Birth Declaration^(30)^. Data for the year 2020 were sourced from the Live Birth Monitoring Panel on the Department of Health Analysis and Non-Communicable Disease Surveillance page^(31)^ due to the unavailability of data on DATASUS. Data were extracted in December 2021 and updated in January 2022. The period from 2014 to 2020 was selected due to the availability of data in the information systems, considering the data collection period and the WHO’s recommendation to utilize the Robson Classification to assess the occurrence of cesareans in health services^(4)^.

### Population and study variables

The population of this study included all women who had either a normal delivery or a cesarean section, based on the analysis of the live birth registry in Brazil, according to records available in these public databases. Initially, the total number of live births for each year of the study was extracted, followed by the selection of the number of births by cesarean surgery and by the 10 groups of the Robson Classification, conducted according to the obstetric characteristics described in Chart 1.

**Chart 1:** Robson Classification according to the obstetric characteristics of each group and the probability of cesarean

Classification	Robson Classification Group Characteristics	Expected Cesarean Rates According to Robson Classification Guidelines
**1**	Nulliparous women with a single cephalic fetus at ≥ 37 weeks in spontaneous labor	Values can be less than 10%
**2**	Nulliparous women with a single cephalic fetus at ≥ 37 weeks, whose labor is induced or who undergo cesarean section before the onset of labor	Between 20-35%
**3**	Multiparous women without previous cesarean, with a single cephalic fetus at ≥ 37 weeks, in spontaneous labor	Usually not exceeding 3.0%
**4**	Multiparous women without previous cesarean, with a single cephalic fetus at ≥ 37 weeks, whose labor is induced or who undergo cesarean section before the onset of labor	Rarely should exceed 15%
**5**	All multiparous women with at least one previous cesarean, with a single cephalic fetus at ≥ 37 weeks	Between 50-60%
**6**	All nulliparous women with a single fetus in breech presentation	Not mentioned in the guidelines
**7**	All multiparous women with a single fetus in breech presentation, including those with previous cesarean(s)	Not mentioned in the guidelines
**8**	All women with multiple gestations, including those with previous cesarean(s)	Close to 60%
**9**	All pregnant women with a fetus in a transverse or oblique lie, including those with previous cesarean(s)	100%
**10**	All pregnant women with a single cephalic fetus at < 37 weeks, including those with previous cesarean(s)	Close to 30%

*Source: based on guidelines from the Pan American Health Organization (PAHO)^(18)^.*

According to the data in Chart 1, groups 1 to 4 consist of nulliparous and multiparous women without prior cesareans, who have a high likelihood of vaginal birth. Group 5 includes multiparous women who have undergone previous cesareans, and groups 6 to 9 comprise women with previous cesareans or nulliparous women with babies in breech, transverse, or oblique positions, as well as women with multiple gestations. Group 10 includes women with a single cephalic fetus at less than 37 weeks, including those with previous cesareans^(18)^.

Regarding the records available from DATASUS, there was a lack of information for the Robson Classification, and thus, these data were extracted as unclassified births, as described in the system^(30)^. Finally, the number of cesareans for each Robson classification group and by type of health establishment, whether public or private, was extracted.

### Data analysis

After extracting the information, the data were tabulated using Excel software. The analysis calculated the following indicators: 1) annual cesarean rate; 2) absolute number and proportion of each Robson group per year, including unclassified births (identified as such); and 3) proportion of cesareans in each Robson group. This analysis was stratified by the type of health establishment where the birth occurred: public or private. It is noted that in this stratification, there were losses of births of less than 1.0%, except in the year 2020 (18,382 - 1.19%).

After calculating the cesarean rate estimates, the Robson classification, and the relationship between the number of these surgeries by Robson group (total, public, and private), a trend analysis was conducted using the Prais-Winsten linear regression model^(32)^. From this model, regression coefficients were estimated, and the annual percentage change (APC) with their respective 95% confidence intervals (CI 95%)^(32)^ was calculated. When the value of the coefficient and the APC is negative, the trend is decreasing; when it is positive, the trend is increasing; and when it is zero, the trend is stable^(32)^. All calculations for the trend analysis were performed using the calculated proportions of the variables.

## RESULTS

During the period from 2014 to 2020, there were 20,298,365 births in the country, averaging 2,899,766.4 births per year, with an average of 1,627,753.14 cesarean births annually. This resulted in an average cesarean rate of 56.1%, with a stable trend over the analyzed period, APC: 0.41 (-1.13 to 1.97), P-trend: 0.55 (data not shown). According to the Robson Classification, the largest proportions of births were classified in the following groups: 5 - women with at least one previous cesarean (average of 21.7% per year); 3 - multiparous women without a previous cesarean (average of 18.8% per year); and 1 - nulliparous women (average of 17.5% per year), in that order. The trend analysis identified increases in the groups: 3 - multiparous women without previous cesarean, with an APC of +4.2 (95% CI +2.5; +5.9); 5 - women with at least one previous cesarean, with an APC of +8.5 (95% CI +6.3; +10.6); and 8 - all women with multiple gestations, with an APC of +2.4 (95% CI +1.5; +3.4) (Table 1). Significant decreasing trends were noted in the groups: 2 - nulliparous women with induced labor or cesarean before the onset of labor, with an APC of -7.5 (95% CI -8.9; -6.1); and 4 - multiparous women with induced labor or cesarean before the onset of labor, with an APC of -5.3 (95% CI -9.2; -1.1). Additionally, it was observed that the number of unclassified births decreased over the period, with an APC of -25.2 (95% CI -32.2; -17.6) (Table 1).

**Table 1:** Number of Live Births in Brazil and Proportion of Births by Robson Classification Group, 2014 to 2020

Classification	2014		2015		2016		2017		2018		2019		2020		Média anual	APC (95% CI)	*p-*trend
	n	%	n	%	n	%	n	%	n	%	n	%	n	%	%		
Robson 1	501,189	16.8	528,855	17.5	511,121	17.8	522,740	17.8	528,341	17.9	502,611	17.6	467,809	17.1	17.5	+0.69 (-2.6; + 4.1)	0.641
Robson 2	483,878	16.2	469,430	15.5	428,708	15.0	423,953	14.5	405,597	13.7	382,943	13.4	365,181	13.4	14.5	-7.5 (-8.9; -6.1)	< 0.0001
Robson 3	524,030	17.5	544,823	18.0	532,690	18.6	553,023	18.9	571,012	19.3	559,600	19.6	531,394	19.4	18.8	+4.2 (+2.5; +5.9)	0.001
Robson 4	302,046	10.1	285,519	9.4	259,570	9.0	258,045	8.8	254,915	8.6	246,682	8.6	239,533	8.7	9.0	-5.3 (-9.2; -1.1)	0.026
Robson 5	574,072	19.2	607,393	20.1	597,353	20.9	639,847	21.8	675,915	22.9	669,360	23.4	644,329	23.6	21.7	+8.5 (+6.3; +10.6)	< 0.0001
Robson 6	42,876	1.4	43,007	1.4	41,290	1.4	40,841	1.4	39,872	1.3	37,282	1.3	34,854	1.2	1.3	-4.6 (-6.7; -2.4)	0.003
Robson 7	52,751	1.7	54,491	1.8	56,684	1.9	56,388	1.9	55,887	1.9	53,706	1.8	51,805	1.9	1.8	+2.2 (-2.0; + 6.7)	0.265
Robson 8	60,019	2.0	61,723	2.0	57,930	2.0	60,335	2.0	62,235	2.1	60,909	2.1	57,983	2.1	2.0	+2.4 (+1.5; +3.4)	0.001
Robson 9	7,858	0.2	7,312	0.2	6,680	0.2	6,088	0.2	6,218	0.2	5,852	0.21	4,912	0.1	0.2	-11.6 (-14.1; -9.1)	< 0.0001
Robson 10	270,783	9.0	264,840	8.78	259,196	9.07	258,705	8.8	261,723	8.8	256,023	8.99	249,623	9.1	8.9	+0.4 (-0.6; +1.5)	0.358
Unclassified	159,757	5.3	150,275	4.98	106,578	3.73	103,570	3.5	83,217	2.8	74,178	2.60	78,602	2.8	3.7	-25.2 (-32.2; -17.6)	0.001

*n - Number; % - Proportion; APC - Annual Percentage Change; 95% CI - 95% Confidence Interval.*

In Table 2, it is observed that the proportions of cesarean sections were high in the Robson groups: 1 - nulliparous women (average of 45.2% per year); 2 - nulliparous women with induced labor or cesarean before the onset of labor (average of 70.3% per year); 4 - multiparous women with induced labor or cesarean before the onset of labor (average of 46.7% per year); and 5 - women with at least one previous cesarean (average of 85.5% per year). Only the Robson 1 group - nulliparous women showed a decrease in the proportion of surgeries during the study period, with an APC of -2.9 (95% CI -5.4; -0.3). The number of unclassified births, including cesarean surgeries, also decreased during the period, with an APC of -3.1 (95% CI -4.3; -2.0). Two groups remained stable: Robson 3 - multiparous women without previous cesarean, with an APC of -3.7 (95% CI -8.7; +1.4), and Robson 5 - women with at least one previous cesarean, with an APC of -0.2 (95% CI -1.1; +0.6). All other groups showed a tendency for an increase in cesareans during the study period, with Robson 4 - multiparous women with induced labor or cesarean before the onset of labor having the highest growth, with an APC of +4.8 (95% CI +3.7; +6.0).

**Table 2:** Number of live births by cesarean and proportion of cesareans by Robson classification group, Brazil, 2014 to 2020

Classification	2014		2015		2016		2017		2018		2019		2020		Média anual	APC (95% CI)	*p-*trend
	n	%	N	%	n	%	n	%	n	%	n	%	n	%	%		
Robson1	244,212	48.7	240,901	45.6	228,966	44.8	232,974	44.6	233,585	44.2	221,145	44.0	207,977	44.6	45.2	-2.93 (-5.47; -0.32)	0.041
Robson 2	336,014	69.4	320,134	68.2	296,668	69.2	295,939	69.8	287,050	70.8	274,569	71.7	266,697	73.0	70.3	+2.32 (+1.26; +3.39)	0.003
Robson 3	112,826	21.5	105,977	19.5	100,478	18.9	102,567	18.5	106,226	18.6	104,084	18.6	101,991	19.2	19.3	-3.79 (-8.76; +1.45)	0.135
Robson 4	134,400	44.5	126,606	44.3	118,867	45.8	118,962	46.1	120,390	47.2	119,456	48.4	120,641	50.4	46.7	+4.88 (+3.75; +6.01)	< 0.0001
Robson 5	497,227	86.6	518,887	85.4	507,756	85.0	544,142	85.0	575,794	85.2	570,374	85.2	553,425	85.9	85.5	-0.25 (-1.10; +0.60)	0.502
Robson 6	38,365	89.5	38,416	89.3	36,952	89.5	36,927	90.4	36,431	91.4	34,267	91.9	31,989	91.8	90.5	+1.23 (+0.70; +1.77)	0.002
Robson 7	44,669	84.7	46,175	84.7	48,691	85.9	49,150	87.2	49,084	87.8	47,517	88.5	45,961	88.7	86.8	+2.06 (+1.58; +2.54)	< 0.0001
Robson 8	49,525	82.5	50,970	82.6	48,243	83.3	50,597	83.9	52,535	84.4	51,470	84.5	49,354	85.1	83.8	+1.28 (+1.08; +1.48)	< 0.0001
Robson 9	7,620	97.0	7,086	96.9	6,476	96.9	5,909	97.1	6,043	97.2	5,699	97.4	4,757	96.8	97.0	+0.15 (+0.05; +0.25)	0.023
Robson 10	137,410	50.7	131,871	49.8	129,970	50.1	131,352	50.8	134,398	51.4	134,699	52.6	134,685	54.0	51.3	+2.58 (+0.66; +4.54)	0.022
Unclassified	95,686	59.9	87,035	57.9	59,886	56.2	58,783	56.8	45,969	55.2	40,909	55.1	42,834	54.5	56.5	-3.17 (-4.32; -2.01)	0.001

*n - Number; % - Proportion; APC - Annual Percentage Change; 95% CI - 95% Confidence Interval.*

**Figure 1:**
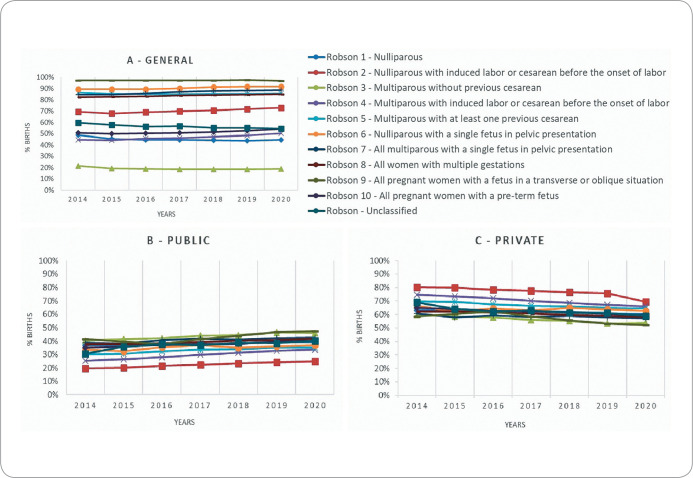
Proportion of live births by cesarean section, by Robson Classification group, stratified by type of health establishment, Brazil, 2014 to 2020

Figure 1 displays the proportion of cesarean sections for each Robson classification, stratified by type of health establishment (public and private). In 2014, 36.8% of cesarean births classified as Robson group 1 (nulliparous women) were performed in the public health sector, while 63.1% took place in private establishments. These proportions are consistently observed throughout the years analyzed and across all Robson groups (refer to Figure 1/Table 3).

**Table 3:** Proportion of live births by cesarean section in Brazil, by Robson classification group and stratified by type of health establishment, 2014 to 2020

Classification	Proporção de Cesáreas Estabelecimentos Públicos									Proporção de Cesáreas Estabelecimentos Privados								
	2014	2015	2016	2017	2018	2019	2020	VMA (IC95%)	*p-trend*	2014	2015	2016	2017	2018	2019	2020	APC (95% CI)	*p-*trend
	%	%	%	%	%	%	%			%	%	%	%	%	%	%		
Robson 1	36.88	36.87	37.16	37.32	37.94	39.30	39.20	+2.73 (+1.46; +4.01)	0.003	63.12	63.13	62.84	62.68	62.01	60.70	60.53	-1.72 (-2.57; -0.87)	0.004
Robson 2	19.74	20.00	21.45	22.31	23.37	24.34	24.89	+10.47 (+9.44; +11.50)	< 0.0001	80.26	80.00	78.54	77.69	76.61	75.65	69.43	-4.20 (-5.83; -2.54)	0.002
Robson 3	40.21	41.50	42.10	44.11	44.64	46.50	45.82	+6.21 (+5.62; +6.80)	< 0.0001	59.79	58.49	57.90	55.88	55.32	53.49	53.84	-4.61 (-4.92; -4.30)	< 0.0001
Robson 4	25.21	26.30	27.89	29.68	31.51	32.91	33.55	+12.16 (+10.06; +14.30)	< 0.0001	74.79	73.70	72.11	70.32	68.47	67.08	66.15	-4.86 (-5.31; -4.42)	< 0.0001
Robson 5	30.16	30.65	32.36	33.61	33.98	35.12	35.19	+6.63 (+4.84; +8.44)	< 0.0001	69.84	69.34	67.64	66.39	65.98	64.87	64.56	-3.21 (-3.78; -2.63)	< 0.0001
Robson 6	33.60	32.65	35.56	36.87	35.24	36.21	37.05	+4.16 (+1.08; +7.34)	0.021	66.40	62.22	64.44	63.13	64.73	63.78	62.75	-0.45 (-1.71; +0.83)	0.427
Robson 7	39.02	37.93	40.58	41.70	40.96	42.16	42.75	+4.31 (+2.51; +6.15)	0.002	60.98	57.69	59.42	58.30	59.00	57.83	57.07	-1.27 (-2.22; -0.30)	0.024
Robson 8	37.75	37.58	38.35	39.29	40.24	40.91	41.78	+4.44 (+3.58; +5.31)	< 0.0001	62.23	62.42	61.64	60.71	59.71	59.09	58.15	-2.82 (-3.39; -2.24)	< 0.0001
Robson 9	41.39	39.50	37.62	41.31	44.28	46.83	47.68	+7.28 (+0.27; +14.77)	0.052	58.61	60.49	62.38	58.66	55.72	53.17	52.07	-5.33 (-9.92; -0.51)	0.043
Robson 10	35.25	35.84	37.17	37.83	38.24	39.52	40.48	+5.38 (+4.82; +5.95)	< 0.0001	64.74	64.16	62.83	62.16	61.73	60.48	59.41	-3.18 (-3.53; -2.82)	< 0.0001
Unclassified	30.49	35.92	37.61	36.96	38.94	38.65	40.49	+8.65 (+3.22; +14.37)	< 0.0001	69.14	64.04	62.17	63.03	61.02	61.34	58.87	-4.53 (-6.71; -2.30)	0.004

*%: Proportion. APC: Annual PercentageChange. 95% CI: 95% Confidence Interval.*

*A - Proportion of cesareans for each classification; B - Proportion for each classification in the public sector; C - Proportion for each classification in the private sector.*

Only Robson Group 9 (all pregnant women with a fetus in a transverse or oblique position) showed stability in the trend analysis among births in public establishments, with an APC of +7.2 (95% CI +0.2 to +14.7) (p-trend=0.052). All other classifications of births in public establishments demonstrated a trend of increasing cesareans, particularly noted in groups: Robson 4 (multiparous women with induced labor or cesarean before the onset of labor), with an APC of +12.1 (95% CI +10.0 to +14.3), and Robson 2 (nulliparous women with induced labor or cesarean before the onset of labor), with an APC of +10.4 (95% CI +9.4 to +11.5). The number of unclassified cases in public establishments also showed an increasing trend, with an APC of +8.6 (95% CI +3.2 to +14.3) (Figure 1/Table 3).

The trend analysis of the proportion of cesareans by Robson groups in private establishments reveals that only Group 6 (nulliparous women with a fetus in breech presentation) demonstrated stability, with an APC of -0.4 (95% CI -1.7 to +0.8), p-trend=0.427. During the study period, all other classifications showed a decreasing trend, with the largest decreases in groups: Robson 9 (all pregnant women with a fetus in a transverse or oblique position), with an APC of -5.3 (95% CI -9.9 to -0.5); Robson 4 (multiparous women with induced labor or cesarean before the onset of labor), with an APC of -4.8 (95% CI -5.3 to -4.4); Robson 3 (multiparous women without a previous cesarean), with an APC of -4.6 (95% CI -4.9 to -4.3); and Robson 2 (nulliparous women with induced labor or cesarean before the onset of labor), with an APC of -4.2 (95% CI -5.8 to -2.5), the latter three groups having a higher likelihood of vaginal birth. The unclassified cases in the private health sector also showed a decreasing trend, with an APC of -4.5 (95% CI -6.7 to -2.3). Despite the observed reduction trend, the private sector still maintains significantly higher proportions of cesareans than the public sector (Figure 1/Table 3).

## DISCUSSION

Findings from this study indicate that the cesarean rate in Brazil has remained endemic over time, consistently exceeding WHO recommendations, corroborating results from previous studies^(20,33)^. Analyzing cesarean trends by Robson Classification groups, it is evident that the proportion of this surgery increased in most groups during the study period, especially those characterized by conditions favorable to vaginal birth in public services. These data highlight the need for health services to utilize updated admission criteria and partogram curves, such as Zhang’s^(34)^, to prevent labor dystocias and the primary cesarean.

In private services, there has been a slight reduction in cesareans, despite the highest rates of surgery occurring in these settings, underscoring the need to maintain efforts to reduce these rates across both types of establishments. One strategy that may have contributed to the reduction of cesareans in the private sector was the implementation of the Adequate Birth Project^(35-36)^, a well-organized strategy developed based on successful experiences in reducing cesareans in the private sector^(35-36)^. This strategy began with 35 institutions and, following the success of the indicators, expanded to 137 institutions^(35-36)^. Comparing one participating institution of this project with other maternity units in the SUS network, participants of the Stork Network, showed improved indicators that point to better use of appropriate technology in labor and birth in the private network^(35)^.

The existing inequalities in the occurrence of cesareans in both public and private sectors, initially favoring women of the SUS, represent a complex situation, as not all women granted vaginal birth recognize it as a benefit. A study that examined how birth experiences are influenced by women’s social class, especially concerning the decision on the mode of birth, since relationships between professionals/services and women are mediated by power in the public system, showed that decisions are usually made by professionals, without significant dialogue with the woman to understand her needs and desires for access to health technologies, such as analgesia^(37)^. In the private system, there is greater attention to women and respect for their choices, allowing them to experience a humanized birth according to their needs, which is not necessarily a natural or demedicalized birth^(37)^.

The increasing trend of cesareans in the SUS is also a cause for alarm, as women experiencing greater vulnerability may face an additional risk: that of an unnecessary cesarean. The best technology, in this case, vaginal birth, becomes available to those who are informed of its benefits and have the social power to make decisions, as well as the choice of a dedicated team to meet individual needs.

Furthermore, the high rates of cesarean sections in the private sector allow for interventions aimed at their reduction, many of which have already been implemented in the public sector^(35)^. To reverse this trend, ongoing public policies in the country need to be continuously monitored to identify effects, make progress, and prevent setbacks. One advancement could be the incorporation of new ways to educate health professionals and the community about the models of care for childbirth and birth in the country, as has been done with the interactive exhibition “Senses of Birth”^(38)^. Lastly, the concept and practice of quaternary prevention may be important aspects in reducing hypermedicalization and preventing iatrogenic effects, including unnecessary cesareans^(39)^.

When analyzing the births that occurred in Brazil during the study period, they were concentrated in Robson groups 1 to 5, accounting for about three-quarters of the total. Monitoring cesareans in these specific groups, the results indicate the maintenance of a scenario already consolidated in the country, even when conditions are favorable for vaginal birth. This is clearly observed in group 1, which, despite showing a decrease over the period, still presents numbers three times higher than the ideal recommended by the guideline^(18)^. A study conducted at a maternity hospital in São Paulo with women from group 1 showed that certain characteristics, such as age and Body Mass Index (BMI) of the mother, directly affect the choice of delivery method, and the main indications for cesarean in these women were: fetal distress (37.4%), cephalopelvic disproportion (37.2%), presence of meconium (8.6%), and suspicion of fetal macrosomia (7.7%)^(40)^. However, these justifications are often described in proportions higher than expected within the indicated cesarean rate.

The study also showed that groups 2 and 4 had a large proportion of cesareans, especially group 2, which, for example, in 2020, showed more than double the expected rate. Moreover, an increasing trend was observed in this group during the analyzed period. Within these groups are women who have induced labors or elective cesareans, i.e., those who would have a great chance of vaginal birth.

However, it is interesting to note that nulliparous women in group 2 have a much higher number of cesareans than multiparous women without previous cesareans (groups 3 and 4). This high number of cesareans in primiparous women is concerning, as it indicates a significant prospect of future cesareans, which could jeopardize maternal health^(41)^ and perpetuate this practice of recommending cesareans in scenarios favorable to vaginal birth, considering the Brazilian context. The findings of this study indicate that despite the implementation of various public policies aimed at humanizing childbirth, such as the Stork Network, CONITEC Guidelines, and programs for Enhancement and Innovation in Obstetric and Neonatal Care, the proportion of cesarean sections in Brazil remains high. This persistence can be particularly attributed to the historical model of technocratic and hypermedicalized obstetric care, characterized by constant unnecessary interventions that prioritize medical knowledge and disregard the woman as the protagonist of her childbirth^(42)^.

To alter this scenario, increasing evidence has demonstrated the benefits of obstetric nursing in the context of childbirth. This evidence shows enhanced satisfaction and empowerment for women throughout the childbirth process, improvements in maternal and neonatal indicators with reductions in non-recommended interventions, a decrease in obstetric violence, and lower cesarean rates^(12-13,42-44)^. Additionally, it contributes to the strengthening of teamwork.

Nurses are involved in the entire care process of labor, delivery, and birth. Therefore, they can significantly contribute to the implementation of public policies, the assurance of women’s and their families’ rights, and the provision of humanized care. Moreover, the education of health professionals, whether in academia or as a continuing presence in health services, must be aligned with theoretical concepts and practices regarding the appropriate use of technology. Notably, the training of obstetric nurses and their effective integration into health services as a strategic public policy are essential to change the care model. The performance of these professionals has led to better outcomes and greater autonomy for women^(12-13,42-44)^, underscoring the importance of nurses in reducing unnecessary interventions during childbirth and in improving maternal and neonatal outcomes.

Despite this recognition and the benefits observed in various countries, the number of professionals remains insufficient for the needs of the services. According to the WHO, the world would need an additional 9 million nurses and midwives to achieve the goal of universal health coverage by 2030^(44)^, thereby enhancing childbirth care and contributing to the reduction of maternal and neonatal mortality. Moreover, the COVID-19 pandemic has impacted all areas of life globally, including imposing many restrictions on women’s rights and good practices in maternity wards^(45)^. In Brazil, in particular, there has been a weakening of policies and a cessation and restriction of investments in the health sector in recent years^(46-47)^, which may contribute to exacerbating problems in obstetric and neonatal care and complicate the achievement of the Sustainable Development Goals (SDGs)^(47)^.

### Study limitations

The primary limitation of this study is the short time period evaluated, which is less ideal for a trend analysis. Despite this, the study presents unprecedented results regarding the trend of cesarean sections according to the Robson classification in both public and private sectors in the country, highlighting the need for increased investments to reduce the occurrence of unnecessary cesareans. It is also worth noting that an appropriate technique for trend analysis in short periods was used and that the Robson Classification was implemented recently as a crucial strategy for continuous monitoring. Another limitation is the unclassified data, which complicates the analysis; however, these have been decreasing over the years, demonstrating a positive development highlighted by this study. This study tracks the rates of cesarean sections by classification, which allowed for assessing the likelihood of normal birth and monitoring these rates over time.

### Contributions to Nursing Field

The main contributions of this study relate to the monitoring of cesarean proportions according to Robson classification groups in a recent period in the country, during which various strategies were implemented to reduce cesarean rates in both public and private sectors, despite recent political-programmatic setbacks. The importance of classifying cesareans according to the Robson classification is underscored as a tool for inducing and assessing the reduction of cesarean rates worldwide. Moreover, this classification allows for comparisons between different locations and services, as demonstrated by the analysis conducted in this study, which revealed differences between public and private services. Although there is a trend of reduction in some groups, they still maintain the highest proportions of cesareans performed in the country. Indirectly, our results show subtle positive and negative changes, pointing to the need to resume and strengthen health policies promoting maternal and infant health in obstetric and neonatal care, including ensuring the training and primarily the performance of obstetric nurses in the childbirth and birth scenarios, beyond their integration into health services.

## CONCLUSIONS

Despite a slight trend toward a reduction in cesarean rates in the private sector, these services continue to exhibit the highest rates in the country. Additionally, there was an increasing trend in the public sector and the maintenance of a high proportion of cesareans among women with conditions favorable for normal birth. In this context, the importance of monitoring these indicators and utilizing the Robson classification as an effective tool to identify the occurrence of unnecessary cesareans is emphasized. Given the observed scenario in the country, there is also a need to strengthen public policies to change the care model and ensure women’s autonomy during labor, delivery, and birth, consequently improving obstetric and neonatal indicators.
