# Supplementary material for: Trends in cesarean section rates in Brazil by Robson classification group, 2014-2020
Source: Rev Bras Enferm. 2024 Jul 29;77(3):e20230099. doi: 10.1590/0034-7167-2023-0099 (PMC11290736; doi:10.1590/0034-7167-2023-0099)
Supplement: Translation

# Translation: Tendência da taxa de cesariana no Brasil por grupo de classificação de Robson, 2014-2020

**DOI:** 10.1590/0034-7167-2023-0099pt

**Authors:** Virginia Barbosa Pereira, Síntia Nascimento dos Reis, Fernanda Gontijo Araújo, Torcata Amorim, Eunice Francisca Martins, Mariana Santos Felisbino-Mendes

**Affiliations:** I Universidade Federal de Minas Gerais. Belo Horizonte, Minas Gerais, Brasil

**Keywords:** Estudos de Séries Temporais, Cesárea, Serviços de Saúde Materna, Saúde Reprodutiva, Enfermagem Obstétrica

## Abstract

**Objetivos:**

avaliar a tendência de cesáreas, no período de 2014 a 2020, nos setores público e privado segundo a Classificação de Robson.

**Métodos:**

estudo de série temporal da proporção de mulheres que tiveram cesáreas entre 2014 e 2020, considerando a classificação de Robson e o tipo de serviço. Para análise de tendência, utilizou-se a regressão de Prais-Winsten.

**Resultados:**

observaram-se maiores proporções de cesáreas em todos os grupos de Robson no setor privado em relação ao público, mesmo com tendência de redução no privado e aumento no público. Também foram registradas elevadas proporções de cesáreas em grupos favoráveis ao parto normal (Robson 1, 4 e 5).

**Conclusões:**

apesar da tendência de redução das cesáreas no setor privado, houve tendência crescente no público e elevada proporção de cesáreas em mulheres com condições favoráveis ao parto normal. Ressalta-se a necessidade de monitorar esses indicadores para avaliar e propor intervenções para a redução de cesáreas desnecessárias.

## INTRODUÇÃO

A cesariana é uma intervenção cirúrgica necessária em casos específicos, como os de descolamento prematuro de placenta, ruptura uterina, sofrimento fetal crônico, entre outras urgências obstétricas, podendo inclusive salvar vidas^(1)^. Contudo, os casos de urgências obstétricas não estão no mesmo nível das taxas dessa cirurgia, visto que a maioria das mulheres apresenta condições saudáveis para o parto vaginal^(2)^. Quando mal indicada, a cesariana pode causar infecções, complicações respiratórias neonatais e aumentar a chance de morte materna e fetal^(2)^. Também está associada a uma alta taxa de prematuridade iatrogênica^(3)^.

A Organização Mundial de Saúde (OMS) recomenda que a taxa deste procedimento não ultrapasse os 15% e, desde 1985, a taxa de cesariana considerada ideal é entre 10 e 15%^(4)^. A proporção média global de cesariana é de 21,1%, variando de 5% na África Subsaariana a 42,8% na América Latina/Caribe^(5)^. Além disso, nas últimas décadas, observou-se um aumento constante nas taxas de cesarianas em todo o mundo, especialmente nos países de baixa e média renda^(6)^. O Brasil tem a segunda maior proporção de cesarianas do mundo (55,7% em 2018), seguido pela República Dominicana (58,1% em 2018)^(7)^. Ressalta-se ainda que quase 90% das cesáreas no Brasil são realizadas entre mulheres que recebem cuidados de saúde durante o parto nos serviços privados^(8)^.

Assim, diante da elevada taxa de cesarianas desnecessárias no país, surgiram ao longo dos anos algumas políticas públicas voltadas para a diminuição dessas taxas e orientadas para o melhor atendimento às gestantes baseado em evidências científicas. Nesse sentido, destacam-se algumas estratégias, tais como o Programa de Humanização no Pré-natal e Nascimento, de 2002^(9)^; a Rede Cegonha, lançada e implementada no Sistema Único de Saúde no ano de 2011^(10)^; e o Programa de Residência em Enfermagem Obstétrica, instituído em 2012^(11)^, que teve como objetivo a capacitação de enfermeiras para atuar na atenção ao pré-natal, ao parto e ao nascimento. Esse profissional tem sido reconhecido pela OMS como capaz de contribuir para a redução de intervenções desnecessárias durante o parto e nascimento, e sua assistência está associada a desfechos maternos e neonatais positivos^(3)^. A atuação dessas enfermeiras também está associada à menor taxa de cesarianas^(12-13)^.

Nesse cenário de crescente fortalecimento das políticas de atenção ao parto e nascimento, em 2016 foram publicadas as Diretrizes de Atenção à Gestante: a operação cesariana, que estabeleceu parâmetros fundamentais para a orientação e avaliação científica e pondera sobre a necessidade (ou não) de realização de cesárea, formulada pela Coordenação Geral de Saúde da Mulher do Ministério da Saúde^(14)^. De forma semelhante, em 2017, a Portaria 353 do Ministério da Saúde estabeleceu as Diretrizes Nacionais de Assistência ao Parto Normal, a fim de que este fosse desenvolvido com máxima segurança e qualidade, tanto para as mulheres quanto para os recém-nascidos^(15)^. Diante desse contexto de mobilização para redução das cesarianas, em 2016, a Agência Nacional de Saúde Suplementar apresentou o Projeto Parto Adequado com mecanismos para redução das cesarianas nas instituições privadas^(16)^.

Uma forma de avaliação das taxas de cesarianas é a Classificação de Robson, proposta pela OMS em 2014. Trata-se de um instrumento padrão que permite comparações entre instituições e países^(4,17)^. A classificação é composta por 10 grupos, mutuamente inclusivos e excludentes, ou seja, toda gestante pode ser classificada em apenas um único grupo, com base em seis características obstétricas prévias e em curso: paridade anterior, cesariana prévia, número de fetos, idade gestacional, início do trabalho de parto e apresentação fetal^(18)^. Assim, essa classificação permite o monitoramento e avaliação das taxas de cesáreas nos diferentes grupos, bem como identificar em quais pode haver melhorias das taxas^(17-18)^.

Os estudos publicados sobre os grupos da Classificação de Robson tratam de aspectos variados. Um estudo realizado na Áustria comparou as mudanças das taxas de cesariana em um hospital universitário após a implementação da Classificação de Robson e mostrou que os maiores contribuintes para a taxa de cesariana eram as mulheres multíparas, com feto a termo e com cesariana anterior^(19)^. Outro estudo avaliou o uso da Classificação de Robson em 21 países com diferentes Índices de Desenvolvimento Humano (IDH), demonstrando que a taxa de cesariana após a indução do parto em mulheres multíparas aumentou significativamente em todos os grupos analisados^(17)^ e foi maior em mulheres com cesárea anterior em países com IDH moderado e baixo^(17)^. No cenário brasileiro, uma pesquisa mostrou que mais de 54% de todas as cesáreas foram realizadas antes do início do trabalho de parto e que, quanto maior o IDH, maior a taxa de cesárea nos grupos socioeconômicos mais vulneráveis^(20)^.

Ainda, outros estudos brasileiros que utilizaram a classificação de Robson foram locais^(21-22)^, restritos a instituições^(23-24)^, ou tiveram outros objetivos como avaliar a associação entre cesarianas e prematuridade^(25)^ ou a associação entre acesso ao pré-natal e ocorrência de cesáreas^(26)^. Outros estudos utilizaram a classificação de Robson para avaliar a ocorrência de cesáreas no país, mas em períodos anteriores, muito próximos da sua implementação enquanto estratégia, tais como de 2011 a 2017^(20)^, e de 2014 a 2016^(27)^. Ainda, um estudo analisou a tendência temporal das taxas de cesáreas entre 1994 e 2019, evidenciando um aumento de 2,1% a cada ano e uma tendência de estabilização a partir de 2012, além de diferenças entre as regiões; porém, este estudo não utilizou a classificação de Robson^(5)^. Assim, destaca-se a relevância de analisar a proporção de cesáreas no país a partir do momento em que a OMS recomendou a classificação de Robson como instrumento de monitoramento e redução das cesáreas^(4)^, bem como de avaliar separadamente os serviços públicos e privados, visto que é observada uma maior proporção de cesáreas nos serviços privados do país^(8)^.

Neste contexto, de alta taxa de cesárias e desigualdades entre grupos socioeconômicos, torna-se importante avaliar como tem evoluído o comportamento das taxas de cesáreas no país, visto que há políticas públicas indutoras para sua redução, tanto nos hospitais públicos quanto nos privados, sendo a classificação de Robson um instrumento importante para o monitoramento da realização de cesáreas desnecessárias.

## OBJETIVOS

Avaliar a tendência de cesáreas, no período de 2014 a 2020, nos setores público e privado segundo a Classificação de Robson.

## MÉTODOS

### Aspectos éticos

Este estudo utilizou dados secundários, agregados, de domínio público, sem a identificação dos sujeitos. Assim, dispensou-se a apreciação do Comitê de Ética, de acordo com a Portaria nº 466, de 12 de dezembro de 2012^(28)^, do Conselho Nacional de Saúde e também o Termo de Consentimento Livre Esclarecido.

### Desenho, período e local do estudo

Trata-se de um estudo ecológico, de série temporal, das taxas de cesáreas nos serviços de saúde públicos e privados do país, segundo a Classificação de Robson, que seguiu as diretrizes STROBE disponíveis na plataforma Equator^(29)^. Foram utilizados dados sobre as gestações e os nascimentos, no período de 2014 a 2019, extraídos do Departamento de Informática do SUS (DATASUS)^(30)^. O Sistema de Informações de Nascidos Vivos (SINASC) é um dos sistemas que alimenta a plataforma do DATASUS e fornece os dados sobre os nascimentos no Brasil, por meio da Declaração de Nascidos Vivos^(30)^. Os dados do ano de 2020 foram extraídos do Painel de Monitoramento de Nascidos Vivos, na página do Departamento de Análise em Saúde e Vigilância das Doenças Não Transmissíveis^(31)^, devido à indisponibilidade dos dados no DATASUS. Os dados foram extraídos em dezembro de 2021 e atualizados em janeiro de 2022. Optou-se por avaliar o período de 2014 a 2020 devido à disponibilidade dos dados nos sistemas de informação, considerando-se o período de coleta de dados e a recomendação da OMS para uso da classificação de Robson para avaliar a ocorrência de cesáreas nos serviços de saúde^(4)^.

### População e variáveis do estudo

A população deste estudo foi constituída por todas as mulheres que tiveram parto normal e cesariana, a partir da análise do registro de nascidos vivos no Brasil, conforme registros disponíveis nessas bases de dados públicas do país. Inicialmente, foi extraído o número total de nascidos vivos em cada ano do estudo, em seguida foi selecionado o número de nascimentos por cirurgia cesariana e pelos 10 grupos da classificação de Robson, realizada conforme características obstétricas descritas no Quadro 1.

**Quadro 1:** Classificação de Robson de acordo com as características obstétricas de cada grupo e a probabilidade de cesariana

Classificação	Características do grupo	% de cesarianas esperadas de acordo com as Diretrizes de Robson
**1**	Nulíparas com feto único, cefálico, ≥ 37 semanas, em trabalho de parto espontâneo	Pode-se alcançar valores inferiores a 10%
**2**	Nulíparas com feto único, cefálico, ≥ 37 semanas, cujo parto é induzido ou que são submetidas à cesárea antes do início do trabalho de parto	Entre 20-35%
**3**	Multíparas sem cesárea anterior, com feto único, cefálico, ≥ 37 semanas, em trabalho de parto espontâneo	Normalmente não superior a 3,0%
**4**	Multíparas sem cesárea anterior, com feto único, cefálico, ≥ 37 semanas, cujo parto é induzido ou que são submetidas à cesárea antes do início do trabalho de parto	Raramente deve ser superior a 15%
**5**	Todas multíparas com pelo menos uma cesárea anterior, com feto único, cefálico, ≥ 37 semanas	Entre 50-60%
**6**	Todas nulíparas com feto único em apresentação pélvica	Sem menção na diretriz
**7**	Todas multíparas com feto único em apresentação pélvica, incluindo aquelas com cesárea(s) anterior(es)	Sem menção na diretriz
**8**	Todas as mulheres com gestação múltipla, incluindo aquelas com cesárea(s) anterior(es)	Perto dos 60%
**9**	Todas as gestantes com feto em situação transversa ou oblíqua, incluindo aquelas com cesárea(s) anterior(es)	100%
**10**	Todas as gestantes com feto único e cefálico, < 37 semanas, incluindo aquelas com cesárea(s) anterior(es)	Perto dos 30%

*Fonte: baseado nas diretrizes da Organização Pan Americana de Saúde (OPAS)^(18)^.*

Conforme os dados constantes no Quadro 1, os grupos de interesse de 1 a 4 indicam nulíparas e multíparas, sem cesárea anterior, que teriam grande probabilidade de parto vaginal. O grupo 5 refere-se a mulheres multíparas que foram submetidas a cesáreas anteriores, e os grupos de 6 a 9 são de mulheres com cesáreas prévias ou nulíparas com bebês em apresentação pélvica, transversa ou oblíqua, além das mulheres com gestação múltipla. Já no grupo 10 estão incluídas mulheres com feto único, cefálico, < 37 semanas, incluindo aquelas com cesáreas anteriores^(18)^.

Em relação ao registro disponível pelo DATASUS, houve ausência de informações para a Classificação de Robson e, assim, essas informações foram extraídas como nascimentos não classificados, conforme descrito no sistema^(30)^. Por fim, foram extraídos o número de cesarianas para cada grupo da classificação de Robson e por tipo de estabelecimento de saúde, públicos ou privados.

### Análise dos dados

Após a extração das informações, o banco de dados foi tabulado no *software Excel*. Na análise, foram calculados os seguintes indicadores: 1) taxa de cesarianas por ano; 2) número absoluto e proporção de cada grupo de Robson por ano, incluindo os nascimentos sem classificação de Robson (identificados como nascimentos não classificados); e 3) proporção de cesarianas em cada grupo de Robson. A mesma análise foi feita estratificando por tipo de estabelecimentos de saúde: público e privado, onde ocorreu o nascimento. Ressalta-se que nessa estratificação houve perdas de nascimentos inferiores a 1,0%, exceto no ano de 2020 (18.382 - 1,19%).

Após calcular as estimativas das taxas de cesariana, a classificação de Robson e a relação entre o número dessas cirurgias por grupo de Robson (total, público e privado), foi realizada a análise de tendência por meio do modelo de regressão linear de Prais-Winsten^(32)^. A partir desse modelo, foram estimados os coeficientes de regressão e calculada a variação média anual (VMA) com seus respectivos intervalos de 95% de confiança (IC 95%)^(32)^. Quando o valor do coeficiente e da VMA é negativo, a tendência é decrescente; quando é positivo, a tendência é crescente; e, quando é igual a 0, a tendência é estacionária^(32)^. Todos os cálculos para a análise de tendência foram realizados com as proporções calculadas das variáveis.

## RESULTADOS

Durante o período de 2014 a 2020, ocorreram 20.298.365 nascimentos no país, com uma média de 2.899.766,4 nascimentos por ano, sendo em média 1.627.753,14 nascimentos por cesariana ao ano, resultando em uma taxa média de 56,1%, com tendência estável durante o período analisado, VMA: 0,41 (-1,13-1,97), *P-trend*: 0,55 (dados não mostrados). Em relação à classificação de Robson, a maior proporção dos nascimentos foi classificada nos grupos: 5 - mulheres com pelo menos uma cesárea anterior (média de 21,7% ao ano); 3 - multíparas sem cesárea anterior (média de 18,8% ao ano); e 1 - nulíparas (média de 17,5% ao ano), nessa ordem. A análise de tendência identificou que houve aumento nos grupos: 3 - multíparas sem cesárea anterior, VMA de +4,2 (IC95% +2,5; +5,9); 5 - mulheres com pelo menos uma cesárea anterior, VMA de +8,5 (IC95% +6,3; +10,6); e 8 - todas as mulheres com gestações múltiplas, VMA de +2,4 (IC95% +1,5; +3,4) (Tabela 1). Houve uma tendência de redução significativa nos grupos: 2 - nulíparas com parto induzido ou cesárea antes do início do trabalho de parto, VMA de -7,5 (IC95% -8,9; -6,1); e 4 - multíparas com parto induzido ou cesárea antes do início do trabalho de parto, VMA de -5,3 (IC95% -9,2; -1,1). Além disso, observou-se que o número de nascimentos não classificados apresentou uma redução no período, VMA de -25,2 (IC95% -32,2; -17,6) (Tabela 1).

**Tabela 1:** Número de nascidos vivos no Brasil e proporção dos nascimentos por classificação de grupo de Robson, 2014 a 2020

Classificação	2014		2015		2016		2017		2018		2019		2020		Média anual	VMA (IC95%)	*p-trend*
	n	%	n	%	n	%	n	%	n	%	n	%	n	%	%		
Robson 1	501.189	16,8	528.855	17,5	511.121	17,8	522.740	17,8	528.341	17,9	502.611	17,6	467.809	17,1	17,5	+0,69 (-2,6; + 4,1)	0,641
Robson 2	483.878	16,2	469.430	15,5	428.708	15,0	423.953	14,5	405.597	13,7	382.943	13,4	365.181	13,4	14,5	-7,5 (-8,9; -6,1)	< 0,0001
Robson 3	524.030	17,5	544.823	18,0	532.690	18,6	553.023	18,9	571.012	19,3	559.600	19,6	531.394	19,4	18,8	+4,2 (+2,5; +5,9)	0,001
Robson 4	302.046	10,1	285.519	9,4	259.570	9,0	258.045	8,8	254.915	8,6	246.682	8,6	239.533	8,7	9,0	-5,3 (-9,2; -1,1)	0,026
Robson 5	574.072	19,2	607.393	20,1	597.353	20,9	639.847	21,8	675.915	22,9	669.360	23,4	644.329	23,6	21,7	+8,5 (+6,3; +10,6)	< 0,0001
Robson 6	42.876	1,4	43.007	1,4	41.290	1,4	40.841	1,4	39.872	1,3	37.282	1,3	34.854	1,2	1,3	-4,6 (-6,7; -2,4)	0,003
Robson 7	52.751	1,7	54.491	1,8	56.684	1,9	56.388	1,9	55.887	1,9	53.706	1,8	51.805	1,9	1,8	+2,2 (-2,0; + 6,7)	0,265
Robson 8	60.019	2,0	61.723	2,0	57.930	2,0	60.335	2,0	62.235	2,1	60.909	2,1	57.983	2,1	2,0	+2,4 (+1,5; +3,4)	0,001
Robson 9	7.858	0,2	7.312	0,2	6.680	0,2	6.088	0,2	6.218	0,2	5.852	0,21	4.912	0,1	0,2	-11,6 (-14,1; -9,1)	< 0,0001
Robson 10	270.783	9,0	264.840	8,78	259.196	9,07	258.705	8,8	261.723	8,8	256.023	8,99	249.623	9,1	8,9	+0,4 (-0,6; +1,5)	0,358
Não classificado	159.757	5,3	150.275	4,98	106.578	3,73	103.570	3,5	83.217	2,8	74.178	2,60	78.602	2,8	3,7	-25,2 (-32,2; -17,6)	0,001

*n - Número, % - Proporção; VMA - Variação Média Anual, IC95% - Intervalo de 95% de confiança.*

Na Tabela 2, observa-se que as proporções de cesarianas foram elevadas nos grupos de Robson 1 - nulíparas (média de 45,2% ao ano); 2 - nulíparas com parto induzido ou cesárea antes do início do trabalho de parto (média de 70,3% ao ano); 4 - multíparas com parto induzido ou cesárea antes do início do trabalho de parto (média de 46,7% ao ano) e 5 - mulheres com pelo menos uma cesárea anterior (média de 85,5% ao ano), e apenas o grupo de Robson 1 - nulíparas apresentou uma diminuição na proporção das cirurgias, durante o período de estudo, com VMA de -2,9 (IC95% -5,4; -0,3). O número de nascimentos não classificados, de cirurgias cesarianas, também diminuiu no período, com VMA de -3,1 (IC95% -4,3; -2,0). Dois grupos se mantiveram estáveis: o Robson 3 - multíparas sem cesárea anterior, com VMA de -3,7 (IC95% -8,7; +1,4), e o Robson 5 - mulheres com pelo menos uma cesárea anterior, com VMA de -0,2 (IC95% -1,1; +0,6). Todos os demais grupos mostraram uma tendência de aumento das cesáreas durante o período do estudo, sendo o grupo Robson 4 - multíparas com parto induzido ou cesárea antes do início do trabalho de parto, o que teve o maior crescimento, com VMA de +4,8 (IC95% +3,7; +6,0).

**Tabela 2:** Número de nascidos vivos por cesarianas e proporção de cesarianas por classificação de grupo de Robson, Brasil, 2014 a 2020

Classificação	2014		2015		2016		2017		2018		2019		2020		Média anual	VMA (IC95%)	*p-trend*
	n	%	N	%	n	%	n	%	n	%	n	%	n	%	%		
Robson1	244.212	48,7	240.901	45,6	228.966	44,8	232.974	44,6	233.585	44,2	221.145	44,0	207.977	44,6	45,2	-2,93 (-5,47; -0,32)	0,041
Robson 2	336.014	69,4	320.134	68,2	296.668	69,2	295.939	69,8	287.050	70,8	274.569	71,7	266.697	73,0	70,3	+2,32 (+1,26; +3,39)	0,003
Robson 3	112.826	21,5	105.977	19,5	100.478	18,9	102.567	18,5	106.226	18,6	104.084	18,6	101.991	19,2	19,3	-3,79 (-8,76; +1,45)	0,135
Robson 4	134.400	44,5	126.606	44,3	118.867	45,8	118.962	46,1	120.390	47,2	119.456	48,4	120.641	50,4	46,7	+4,88 (+3,75; +6,01)	< 0,0001
Robson 5	497.227	86,6	518.887	85,4	507.756	85,0	544.142	85,0	575.794	85,2	570.374	85,2	553.425	85,9	85,5	-0,25 (-1,10; +0,60)	0,502
Robson 6	38.365	89,5	38.416	89,3	36.952	89,5	36.927	90,4	36.431	91,4	34.267	91,9	31.989	91,8	90,5	+1,23 (+0,70; +1,77)	0,002
Robson 7	44.669	84,7	46.175	84,7	48.691	85,9	49.150	87,2	49.084	87,8	47.517	88,5	45.961	88,7	86,8	+2,06 (+1,58; +2,54)	< 0,0001
Robson 8	49.525	82,5	50.970	82,6	48.243	83,3	50.597	83,9	52.535	84,4	51.470	84,5	49.354	85,1	83,8	+1,28 (+1,08; +1,48)	< 0,0001
Robson 9	7.620	97,0	7.086	96,9	6.476	96,9	5.909	97,1	6.043	97,2	5.699	97,4	4.757	96,8	97,0	+0,15 (+0,05; +0,25)	0,023
Robson 10	137.410	50,7	131.871	49,8	129.970	50,1	131.352	50,8	134.398	51,4	134.699	52,6	134.685	54,0	51,3	+2,58 (+0,66; +4,54)	0,022
Não classificado	95.686	59,9	87.035	57,9	59.886	56,2	58.783	56,8	45.969	55,2	40.909	55,1	42.834	54,5	56,5	-3,17 (-4,32; -2,01)	0,001

*n - Número; % - Proporção; VMA - Variação Média Anual; IC95% - Intervalo de 95% de confiança.*

A Figura 1 mostra a proporção de cesarianas por cada classificação de Robson, estratificada por tipo de estabelecimento de saúde (públicos e privados). No ano de 2014, 36,8% dos nascimentos por cesáreas classificados como grupo 1 de Robson (nulíparas) foram realizados no setor público de saúde, enquanto 63,1% ocorreram em estabelecimentos privados. Essas proporções se repetem consecutivamente durante os anos analisados e em todos os grupos de Robson (Figura 1/Tabela 3).

**Tabela 3:** Proporção de nascidos vivos por cesárea no Brasil, por cada classificação de grupo de Robson e estratificado por tipo de estabelecimento de saúde, 2014 a 2020

Classificação	Proporção de Cesáreas Estabelecimentos Públicos									Proporção de Cesáreas Estabelecimentos Privados								
	2014	2015	2016	2017	2018	2019	2020	VMA (IC95%)	*p-trend*	2014	2015	2016	2017	2018	2019	2020	VMA (IC95%)	*p-trend*
	%	%	%	%	%	%	%			%	%	%	%	%	%	%		
Robson 1	36,88	36,87	37,16	37,32	37,94	39,30	39,20	+2,73 (+1,46; +4,01)	0,003	63,12	63,13	62,84	62,68	62,01	60,70	60,53	-1,72 (-2,57; -0,87)	0,004
Robson 2	19,74	20,00	21,45	22,31	23,37	24,34	24,89	+10,47 (+9,44; +11,50)	< 0,0001	80,26	80,00	78,54	77,69	76,61	75,65	69,43	-4,20 (-5,83; -2,54)	0,002
Robson 3	40,21	41,50	42,10	44,11	44,64	46,50	45,82	+6,21 (+5,62; +6,80)	< 0,0001	59,79	58,49	57,90	55,88	55,32	53,49	53,84	-4,61 (-4,92; -4,30)	< 0,0001
Robson 4	25,21	26,30	27,89	29,68	31,51	32,91	33,55	+12,16 (+10,06; +14,30)	< 0,0001	74,79	73,70	72,11	70,32	68,47	67,08	66,15	-4,86 (-5,31; -4,42)	< 0,0001
Robson 5	30,16	30,65	32,36	33,61	33,98	35,12	35,19	+6,63 (+4,84; +8,44)	< 0,0001	69,84	69,34	67,64	66,39	65,98	64,87	64,56	-3,21 (-3,78; -2,63)	< 0,0001
Robson 6	33,60	32,65	35,56	36,87	35,24	36,21	37,05	+4,16 (+1,08; +7,34)	0,021	66,40	62,22	64,44	63,13	64,73	63,78	62,75	-0,45 (-1,71; +0,83)	0,427
Robson 7	39,02	37,93	40,58	41,70	40,96	42,16	42,75	+4,31 (+2,51; +6,15)	0,002	60,98	57,69	59,42	58,30	59,00	57,83	57,07	-1,27 (-2,22; -0,30)	0,024
Robson 8	37,75	37,58	38,35	39,29	40,24	40,91	41,78	+4,44 (+3,58; +5,31)	< 0,0001	62,23	62,42	61,64	60,71	59,71	59,09	58,15	-2,82 (-3,39; -2,24)	< 0,0001
Robson 9	41,39	39,50	37,62	41,31	44,28	46,83	47,68	+7,28 (+0,27; +14,77)	0,052	58,61	60,49	62,38	58,66	55,72	53,17	52,07	-5,33 (-9,92; -0,51)	0,043
Robson 10	35,25	35,84	37,17	37,83	38,24	39,52	40,48	+5,38 (+4,82; +5,95)	< 0,0001	64,74	64,16	62,83	62,16	61,73	60,48	59,41	-3,18 (-3,53; -2,82)	< 0,0001
Não Classificado	30,49	35,92	37,61	36,96	38,94	38,65	40,49	+8,65 (+3,22; +14,37)	< 0,0001	69,14	64,04	62,17	63,03	61,02	61,34	58,87	-4,53 (-6,71; -2,30)	0,004

*% - Proporção; VMA - Variação Média Anual; IC95% - Intervalo de 95% de confiança.*

**Figura 1:** Proporção de nascidos vivos por cesárea, por grupo de Classificação de Robson, estratificado por tipo de estabelecimento de saúde, Brasil, 2014 a 2020

*A - Proporção das cesarianas por cada classificação; B - proporção por cada classificação no setor público; C - proporção por cada classificação no setor privado.*

Apenas o grupo 9 de Robson (todas as gestantes com feto em situação transversa ou oblíqua) mostrou estabilidade na análise de tendência entre os nascimentos ocorridos nos estabelecimentos públicos, com VMA de +7,2 (IC95% +0,2; +14,7) (*p-trend*= 0,052). Todas as demais classificações dos nascimentos ocorridos nos estabelecimentos públicos apresentaram tendência de aumento das cesarianas, destacando-se os grupos: Robson 4 (multíparas com parto induzido ou cesárea antes do início do trabalho de parto), com VMA de +12,1 (IC95% +10,0; +14,3) e o Robson 2 (nulíparas com parto induzido ou cesárea antes do início do trabalho de parto), com VMA de +10,4 (IC95% +9,4; +11,5). O número de não classificações nos estabelecimentos públicos também apresentou tendência de aumento, com VMA de +8,6 (IC95% +3,2; +14,3) (Figura 1/Tabela 3).

A análise de tendência da proporção de cesáreas por grupos de Robson nos estabelecimentos privados mostra que apenas o grupo 6 (nulíparas com feto em apresentação pélvica) demonstrou estabilidade, com VMA de -0,4 (IC95% -1,7; +0,8), *p-trend*=0,427. Durante o período do estudo, todas as outras classificações apresentaram tendência decrescente, com as maiores diminuições nos grupos: Robson 9 (todas as gestantes com feto em situação transversa ou oblíqua), com VMA de -5,3 (IC95% -9,9; -0,5); Robson 4 (multíparas com parto induzido ou cesárea antes do início do trabalho de parto), com VMA de -4,8 (IC95% -5,3; -4,4); Robson 3 (multíparas sem cesárea anterior), com VMA de -4,6 (IC95% -4,9; -4,3); e Robson 2 (nulíparas com parto induzido ou cesárea antes do início do trabalho de parto), com VMA de -4,2 (IC95% -5,8; -2,5), sendo essas três últimas classificações com maior probabilidade de parto vaginal. As não classificações no setor privado de saúde apresentaram tendência de diminuição, com VMA de -4,5 (IC95% -6,7; -2,3). Apesar da tendência de redução observada, o setor privado ainda tem proporções muito maiores de cesáreas do que o setor público (Figura 1/Tabela 3).

## DISCUSSÃO

Os achados desta pesquisa mostram que a taxa de cesariana no Brasil, ao longo do tempo, tem se mantido endêmica, e seus valores permanecem muito acima das recomendações da OMS, corroborando os resultados de estudos prévios^(20,33)^. Ao analisar as tendências das cesarianas por grupos da Classificação de Robson, constata-se que a proporção dessa cirurgia foi crescente na maioria dos grupos no período estudado, principalmente naqueles que caracterizam condições favoráveis ao parto vaginal nos serviços públicos. Esses dados mostram a necessidade de os serviços de saúde utilizarem critérios de internação e curvas de partogramas mais atualizados, como o de Zhang^(34)^, para a prevenção das distocias de trabalho de parto e para a prevenção da primeira cesárea.

Em relação aos serviços privados, observa-se uma redução discreta das cesarianas, apesar de ocorrerem as maiores taxas da cirurgia nesses serviços, evidenciando-se a necessidade da manutenção da mobilização para a redução dessas taxas em ambos os tipos de estabelecimentos. Uma estratégia que pode ter contribuído para a redução das cesáreas no setor privado foi a implementação do PPA^(35-36)^, uma estratégia bem organizada e desenvolvida com base em experiências bem-sucedidas de redução de cesarianas no setor privado^(35-36)^. O PPA teve seu início com 35 instituições e, após o sucesso dos indicadores, foi ampliado para 137 instituições^(35-36)^. Ao comparar uma instituição participante desse projeto com outras maternidades da rede SUS, participantes da Rede Cegonha, foi constatada elevação dos indicadores que apontam para a melhoria do uso de tecnologia apropriada no trabalho de parto e nascimento na rede privada^(35)^.

As desigualdades existentes na ocorrência das cesarianas no setor público e privado, a princípio privilegiando as mulheres do SUS, representam uma situação complexa, pois nem sempre essas mulheres contempladas com o parto vaginal reconhecem-no como um benefício. Um estudo que avaliou como as experiências de parto são influenciadas pela classe social da mulher, especialmente no tocante à decisão da via de parto, visto que as relações entre os profissionais/serviços e as mulheres são mediadas pelo poder no sistema público, mostrou que as decisões ficam, usualmente, a cargo dos profissionais, sem maior diálogo com a mulher para entender suas necessidades e anseios de acesso a tecnologias de saúde, como a analgesia^(37)^. Já no sistema privado, há uma maior escuta das mulheres e respeito às escolhas, de forma que vivenciem uma experiência de parto humanizado de acordo com suas necessidades, o que não necessariamente é um parto natural ou desmedicalizado^(37)^.

A tendência de aumento das cesarianas no SUS também é motivo de alerta, pois as mulheres que vivenciam situações de maior vulnerabilidade podem enfrentar mais um risco: o da cesariana desnecessária. A melhor tecnologia, no caso o parto vaginal, passa a ser usufruída por quem tem a informação de seus benefícios e o poder de classe para a tomada de decisão, assim como a escolha de uma equipe própria para atender às necessidades individuais.

Ademais, as altas taxas de cesarianas no setor privado permitem intervenções para sua redução, muitas já implementadas no setor público^(35)^. Para reverter esse cenário, as políticas públicas, já em curso no país, precisam ser monitoradas continuamente para identificar efeitos, avanços e evitar retrocessos. Um avanço pode ser a incorporação de novas formas de educar profissionais de saúde e a comunidade para os modelos de atenção ao parto e nascimento no país, como tem feito a exposição interativa “Sentidos do Nascer”^(38)^. Por fim, o conceito e a prática de prevenção quaternária podem ser aspectos importantes para redução da hipermedicalização e prevenção das iatrogenias, incluindo as cesarianas desnecessárias^(39)^.

Quando avaliados os nascimentos ocorridos no Brasil durante o período do estudo, estes se concentram nos grupos de Robson de 1 a 5, chegando a somar cerca de três quartos do total. Ao monitorar as cesarianas nesses grupos especificamente, os resultados indicam a manutenção de um cenário já consolidado no país, mesmo quando há condições favoráveis à realização do parto vaginal. Isso pode ser observado claramente no grupo 1 que, apesar de mostrar uma diminuição ao longo do período, ainda apresenta números três vezes maiores de cesarianas do que o ideal recomendado pela diretriz^(18)^. Estudo realizado em uma maternidade de São Paulo com as mulheres do grupo 1 mostrou que algumas características, como a idade e o Índice de Massa Corporal (IMC) da mãe, afetam diretamente a escolha da via de parto e que as principais indicações de cesariana nessas mulheres foram: sofrimento fetal (37,4%), desproporção cefalopélvica (37,2%), presença de mecônio (8,6%) e suspeita de macrossomia fetal (7,7%)^(40)^. Contudo, essas justificativas são frequentemente descritas em proporções superiores às esperadas dentro da taxa de cesariana indicada.

O presente estudo mostrou também que os grupos 2 e 4 apresentaram uma grande proporção de cesarianas, principalmente o grupo 2, visto que, no ano de 2020, por exemplo, apresentou mais do que o dobro da taxa esperada. Além disso, nesse grupo, observou-se uma tendência de aumento durante o período analisado. Dentro desses grupos estão as mulheres que têm seus partos induzidos ou ainda que fazem cesarianas eletivas, ou seja, são condizentes com mulheres que teriam grande chance de um parto vaginal.

No entanto, é interessante perceber que as mulheres nulíparas do grupo 2 têm um número muito maior de cesarianas do que as multíparas sem cesárea anterior (grupos 3 e 4). Esse alto número de cesarianas em mulheres primíparas é preocupante, uma vez que indica uma grande perspectiva de futuras cesarianas, o que pode colocar em risco a saúde materna^(41)^ e perpetuar essa prática de indicação de cesarianas em cenários favoráveis ao parto vaginal, visto o cenário brasileiro.

Os achados deste estudo também apontam que, mesmo após a implementação de diversas políticas públicas que prezam pela humanização do parto, como a Rede Cegonha, as Diretrizes da CONITEC, e o Aprimoramento e Inovação no Cuidado e Ensino em Obstetrícia e Neonatologia, a proporção de cesáreas no Brasil continua elevada. Pode-se inferir que isso se deve particularmente ao histórico modelo de assistência obstétrica tecnocrata e hipermedicalizado, marcado por constantes intervenções desnecessárias, visando o saber médico, e que desconsidera a mulher como protagonista do seu parto^(42)^.

Para mudar esse cenário, evidências crescentes têm mostrado os benefícios da atuação da enfermagem obstétrica no contexto do parto, demonstrando aumento da satisfação e empoderamento da mulher durante todo o processo de parturição, melhora dos indicadores maternos e neonatais com as reduções de intervenções não recomendadas, redução da violência obstétrica e das taxas de cesárea^(12-13,42-44)^, além de contribuir para o fortalecimento do trabalho em equipe.

O enfermeiro participa de todo o processo assistencial do trabalho de parto, parto e nascimento. Portanto, pode contribuir para a efetivação das políticas públicas, da garantia dos direitos das mulheres e suas famílias, bem como do cuidado humanizado. Ademais, a formação dos profissionais de saúde, seja na academia ou de forma permanente nos serviços de saúde, deve estar alinhada com os conceitos teóricos e práticas sobre o uso da tecnologia de forma apropriada. Destaca-se ainda a formação de enfermeiras obstétricas e sua efetiva inserção nos serviços de saúde enquanto política pública estratégica que se faz necessária para mudança do modelo de assistência. A atuação dessas profissionais tem garantido melhores desfechos e maior autonomia das mulheres^(12-13,42-44)^. Assim, ressalta-se a importância das enfermeiras para a redução das intervenções desnecessárias na cena do parto e nascimento, além de melhores desfechos maternos e neonatais.

Apesar deste reconhecimento e benefícios em diversos países, o número de profissionais é insuficiente para os serviços. Segundo a OMS, o mundo precisaria de mais 9 milhões de enfermeiras(os) e parteiras para atingir a meta de cobertura universal de saúde até 2030^(44)^ e, desta forma, melhorar a assistência ao parto, contribuindo com a redução das mortalidades materna e neonatal. Soma-se a isso, o cenário vivenciado no país e no mundo decorrente da pandemia da covid-19, a qual impactou todas as áreas da vida, incluindo muitas restrições de direitos das mulheres e boas práticas nas maternidades^(45)^. No Brasil, de forma peculiar, houve enfraquecimento das políticas e interrupção e restrição de investimentos no setor saúde nos últimos anos^(46-47)^, o que pode contribuir para o agravamento dos problemas da atenção obstétrica e neonatal e à dificuldade no alcance dos Objetivos de Desenvolvimento Sustentável (ODS)^(47)^.

### Limitações do estudo

A principal limitação deste estudo refere-se ao curto período de tempo avaliado para um estudo de tendência. Apesar disso, este estudo apresenta resultados inéditos sobre a tendência das cesarianas segundo a classificação de Robson nos serviços públicos e privados do país, evidenciando a necessidade de mais investimentos para a redução da ocorrência das cesáreas desnecessárias. Vale ressaltar também que foi utilizada uma técnica apropriada para análise de tendência em períodos curtos e que a Classificação de Robson foi implementada recentemente, como uma importante estratégia de monitoramento constante. Outra limitação são os dados não classificados, que dificultam a análise; entretanto, eles vêm diminuindo ao longo dos anos, como um ponto positivo demonstrado por este estudo. Esse estudo mostra o monitoramento das taxas por classificação, ou seja, por características que permitiram avaliar a possibilidade do parto normal e monitorar essas taxas ao longo do tempo.

### Contribuições para a Área da Enfermagem

As principais contribuições deste estudo referem-se ao monitoramento da proporção de cesáreas segundo a classificação dos grupos de Robson em um período recente no país, no qual diversas estratégias foram implementadas para a redução de cesáreas, tanto nos serviços públicos como privados, apesar de retrocessos político-programáticos mais recentes nessa área. Destaca-se ainda a importância da classificação das cesáreas segundo a classificação de Robson, uma ferramenta indutora e de avaliação da redução das taxas de cesarianas em todo o mundo. Além disso, permite a comparação entre diferentes locais e serviços, conforme a análise proposta neste estudo, que mostrou diferenças entre os serviços públicos e privados, que, apesar de apresentar tendência de redução em alguns grupos, ainda têm as maiores proporções de cesárias realizadas no país. De forma indireta, nossos resultados mostram mudanças discretas positivas e negativas, o que aponta para a necessidade de retomar e fortalecer as políticas promotoras da saúde materno-infantil na assistência obstétrica e neonatal, incluindo a garantia da formação e, principalmente, atuação das enfermeiras obstétricas no cenário do parto e nascimento, para além da sua inserção nos serviços de saúde.

## CONCLUSÕES

Apesar da discreta tendência de redução das taxas de cesáreas no setor privado, esses serviços apresentam as maiores taxas no país. Além disso, houve tendência crescente no setor público e a manutenção de uma elevada proporção de cesarianas em mulheres com condições favoráveis para o parto normal. Nesse sentido, destaca-se a importância do monitoramento desses indicadores, e a classificação de Robson como um instrumento eficaz para identificar a ocorrência de cesarianas desnecessárias. Diante do cenário observado no país, tem-se ainda a necessidade de fortalecimento das políticas públicas para a mudança do modelo de assistência e a garantia da autonomia das mulheres durante o trabalho de parto, parto e nascimento e consequente melhoria dos indicadores obstétricos e neonatais.
